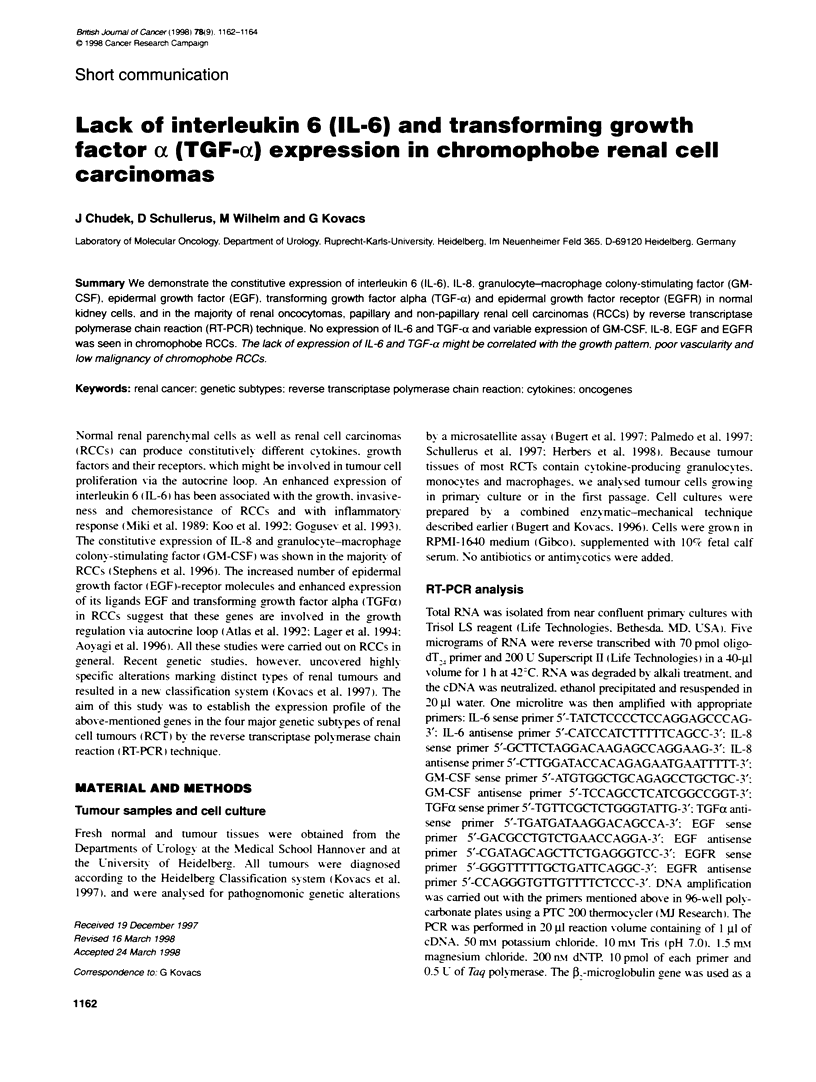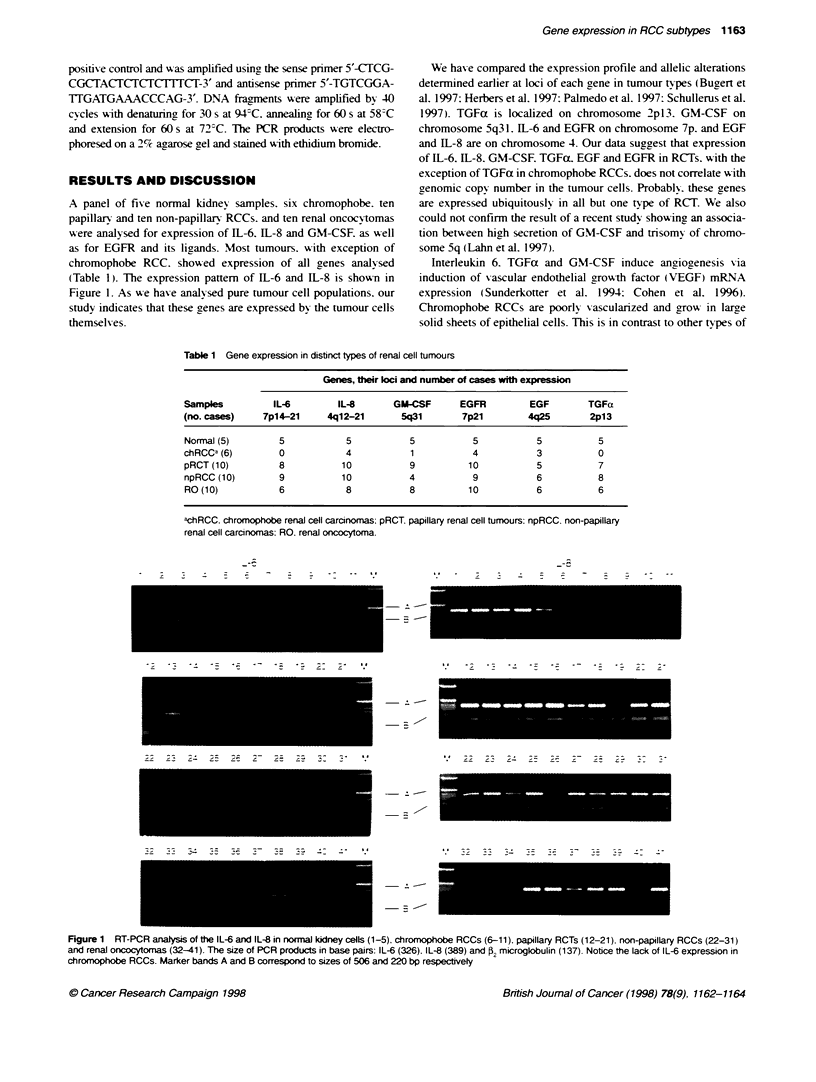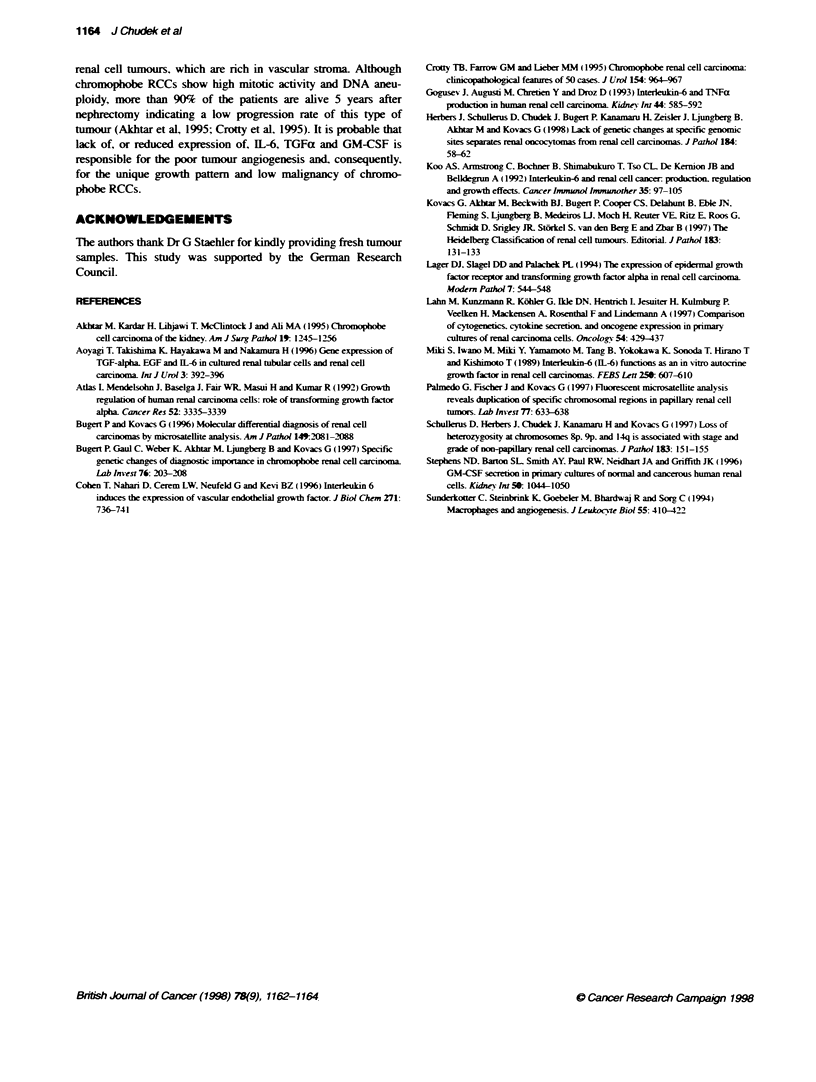# Lack of interleukin 6 (IL-6) and transforming growth factor alpha (TGF-alpha) expression in chromophobe renal cell carcinomas.

**DOI:** 10.1038/bjc.1998.647

**Published:** 1998-11

**Authors:** J. Chudek, D. Schullerus, M. Wilhelm, G. Kovacs

**Affiliations:** Department of Urology, Ruprecht-Karls-University, Heidelberg, Germany.

## Abstract

**Images:**


British Journal of Cancer (1998) 78(9), 1162–1164

© 1998 Cancer Research Campaign

Short communication

### Lack of interleukin 6 (IL-6) and transforming growth factor α (TGF-α) expression in chromophobe renal cell carcinomas

**J Chudek, D Schullerus, M Wilhelm and G Kovacs**

Laboratory of Molecular Oncology, Department of Urology, Ruprecht-Karls-University, Heidelberg, Im Neuenheimer Feld 365, D-69120 Heidelberg, Germany

**Summary** We demonstrate the constitutive expression of interleukin 6 (IL-6), IL-8, granulocyte–macrophage colony-stimulating factor (GM-CSF), epidermal growth factor (EGF), transforming growth factor alpha (TGF-α) and epidermal growth factor receptor (EGFR) in normal kidney cells, and in the majority of renal oncocytomas, papillary and non-papillary renal cell carcinomas (RCCs) by reverse transcriptase polymerase chain reaction (RT-PCR) technique. No expression of IL-6 and TGF-α and variable expression of GM-CSF, IL-8, EGF and EGFR was seen in chromophobe RCCs. *The lack of expression of IL-6 and TGF-α might be correlated with the growth pattern, poor vascularity and low malignancy of chromophobe RCCs.*

**Keywords:** renal cancer; genetic subtypes; reverse transcriptase polymerase chain reaction; cytokines; oncogenes

Normal renal parenchymal cells as well as renal cell carcinomas (RCCs) can produce constitutively different cytokines, growth factors and their receptors, which might be involved in tumour cell proliferation via the autocrine loop. An enhanced expression of interleukin 6 (IL-6) has been associated with the growth, invasiveness and chemoresistance of RCCs and with inflammatory response (Miki et al, 1989; Koo et al, 1992; Gogusev et al, 1993). The constitutive expression of IL-8 and granulocyte–macrophage colony-stimulating factor (GM-CSF) was shown in the majority of RCCs (Stephens et al, 1996). The increased number of epidermal growth factor (EGF)-receptor molecules and enhanced expression of its ligands EGF and transforming growth factor alpha (TGFα) in RCCs suggest that these genes are involved in the growth regulation via autocrine loop (Atlas et al, 1992; Lager et al, 1994; Aoyagi et al, 1996). All these studies were carried out on RCCs in general. Recent genetic studies, however, uncovered highly specific alterations marking distinct types of renal tumours and resulted in a new classification system (Kovacs et al, 1997). The aim of this study was to establish the expression profile of the above-mentioned genes in the four major genetic subtypes of renal cell tumours (RCT) by the reverse transcriptase polymerase chain reaction (RT-PCR) technique.

### MATERIAL AND METHODS

### Tumour samples and cell culture

Fresh normal and tumour tissues were obtained from the Departments of Urology at the Medical School Hannover and at the University of Heidelberg. All tumours were diagnosed according to the Heidelberg Classification system (Kovacs et al, 1997), and were analysed for pathognomonic genetic alterations by a microsatellite assay (Bugert et al, 1997; Palmedo et al, 1997; Schullerus et al, 1997; Herbers et al, 1998). Because tumour tissues of most RCTs contain cytokine-producing granulocytes, monocytes and macrophages, we analysed tumour cells growing in primary culture or in the first passage. Cell cultures were prepared by a combined enzymatic–mechanical technique described earlier (Bugert and Kovacs, 1996). Cells were grown in RPMI-1640 medium (Gibco), supplemented with 10% fetal calf serum. No antibiotics or antimycotics were added.

*Received 19 December 1997*
*Revised 16 March 1998*
*Accepted 24 March 1998*

*Correspondence to:* G Kovacs

### RT-PCR analysis

Total RNA was isolated from near confluent primary cultures with Trisol LS reagent (Life Technologies, Bethesda, MD, USA). Five micrograms of RNA were reverse transcribed with 70 pmol oligo-$dT_{24}$ primer and 200 U Superscript II (Life Technologies) in a 40-μl volume for 1 h at 42°C. RNA was degraded by alkali treatment, and the cDNA was neutralized, ethanol precipitated and resuspended in 20 μl water. One microlitre was then amplified with appropriate primers: IL-6 sense primer 5′-TATCTCCCCTCCAGGAGCCCAG-3′; IL-6 antisense primer 5′-CATCCATCTTTTTCAGCC-3′; IL-8 sense primer 5′-GCTTCTAGGACAAGAGCCAGGAAG-3′; IL-8 antisense primer 5′-CTTGGATACCACAGAGAATGAATTTTT-3′; GM-CSF sense primer 5′-ATGTGGCTGCAGAGCCTGCTGC-3′; GM-CSF antisense primer 5′-TCCAGCCTCATCGGCCGGT-3′; TGFα sense primer 5′-TGTTCGCTCTGGGTATTG-3′; TGFα antisense primer 5′-TGATGATAAGGACAGCCA-3′; EGF sense primer 5′-GACGCCTGTCTGAACCAGGA-3′; EGF antisense primer 5′-CGATAGCAGCTTCTGAGGGTCC-3′; EGFR sense primer 5′-GGGTTTTTGCTGATTCAGGC-3′; EGFR antisense primer 5′-CCAGGGTGTTGTTTTCTCCC-3′. DNA amplification was carried out with the primers mentioned above in 96-well polycarbonate plates using a PTC 200 thermocycler (MJ Research). The PCR was performed in 20 μl reaction volume containing of 1 μl of cDNA, 50 mM potassium chloride, 10 mM Tris (pH 7.0), 1.5 mM magnesium chloride, 200 nM dNTP, 10 pmol of each primer and 0.5 U of *Taq* polymerase. The $\beta_2$-microglobulin gene was used as a positive control and was amplified using the sense primer 5′-CTCG-CGCTACTCTCTCTTTCT-3′ and antisense primer 5′-TGTCGGA-TTGATGAAACCCAG-3′. DNA fragments were amplified by 40 cycles with denaturing for 30 s at 94°C, annealing for 60 s at 58°C and extension for 60 s at 72°C. The PCR products were electrophoresed on a 2% agarose gel and stained with ethidium bromide.

### RESULTS AND DISCUSSION

A panel of five normal kidney samples, six chromophobe, ten papillary and ten non-papillary RCCs, and ten renal oncocytomas were analysed for expression of IL-6, IL-8 and GM-CSF, as well as for EGFR and its ligands. Most tumours, with exception of chromophobe RCC, showed expression of all genes analysed (Table 1). The expression pattern of IL-6 and IL-8 is shown in Figure 1. As we have analysed pure tumour cell populations, our study indicates that these genes are expressed by the tumour cells themselves.

We have compared the expression profile and allelic alterations determined earlier at loci of each gene in tumour types (Bugert et al, 1997; Herbers et al, 1997; Palmedo et al, 1997; Schullerus et al, 1997). TGFα is localized on chromosome 2p13, GM-CSF on chromosome 5q31, IL-6 and EGFR on chromosome 7p, and EGF and IL-8 are on chromosome 4. Our data suggest that expression of IL-6, IL-8, GM-CSF, TGFα, EGF and EGFR in RCTs, with the exception of TGFα in chromophobe RCCs, does not correlate with genomic copy number in the tumour cells. Probably, these genes are expressed ubiquitously in all but one type of RCT. We also could not confirm the result of a recent study showing an association between high secretion of GM-CSF and trisomy of chromosome 5q (Lahn et al, 1997).

Interleukin 6, TGFα and GM-CSF induce angiogenesis via induction of vascular endothelial growth factor (VEGF) mRNA expression (Sunderkotter et al, 1994; Cohen et al, 1996). Chromophobe RCCs are poorly vascularized and grow in large solid sheets of epithelial cells. This is in contrast to other types of

**Table 1** Gene expression in distinct types of renal cell tumours

| | Genes, their loci and number of cases with expression | | | | | |
|---|---|---|---|---|---|---|
| **Samples (no. cases)** | **IL-6 7p14–21** | **IL-8 4q12–21** | **GM-CSF 5q31** | **EGFR 7p21** | **EGF 4q25** | **TGFα 2p13** |
| Normal (5) | 5 | 5 | 5 | 5 | 5 | 5 |
| chRCC[a] (6) | 0 | 4 | 1 | 4 | 3 | 0 |
| pRCT (10) | 8 | 10 | 9 | 10 | 5 | 7 |
| npRCC (10) | 9 | 10 | 4 | 9 | 6 | 8 |
| RO (10) | 6 | 8 | 8 | 10 | 6 | 6 |

[a]chRCC, chromophobe renal cell carcinomas; pRCT, papillary renal cell tumours; npRCC, non-papillary renal cell carcinomas; RO, renal oncocytoma.

**Figure 1** RT-PCR analysis of the IL-6 and IL-8 in normal kidney cells (1–5), chromophobe RCCs (6–11), papillary RCTs (12–21), non-papillary RCCs (22–31) and renal oncocytomas (32–41). The size of PCR products in base pairs: IL-6 (326), IL-8 (389) and $\beta_2$ microglobulin (137). Notice the lack of IL-6 expression in chromophobe RCCs. Marker bands A and B correspond to sizes of 506 and 220 bp respectively

© Cancer Research Campaign 1998

renal cell tumours, which are rich in vascular stroma. Although chromophobe RCCs show high mitotic activity and DNA aneuploidy, more than 90% of the patients are alive 5 years after nephrectomy indicating a low progression rate of this type of tumour (Akhtar et al, 1995; Crotty et al, 1995). It is probable that lack of, or reduced expression of, IL-6, TGFα and GM-CSF is responsible for the poor tumour angiogenesis and, consequently, for the unique growth pattern and low malignancy of chromophobe RCCs.

### ACKNOWLEDGEMENTS

The authors thank Dr G Staehler for kindly providing fresh tumour samples. This study was supported by the German Research Council.

### REFERENCES

Akhtar M, Kardar H, Lihjawi T, McClintock J and Ali MA (1995) Chromophobe cell carcinoma of the kidney. *Am J Surg Pathol* **19**: 1245–1256

Aoyagi T, Takishima K, Hayakawa M and Nakamura H (1996) Gene expression of TGF-alpha, EGF and IL-6 in cultured renal tubular cells and renal cell carcinoma. *Int J Urol* **3**: 392–396

Atlas I, Mendelsohn J, Baselga J, Fair WR, Masui H and Kumar R (1992) Growth regulation of human renal carcinoma cells: role of transforming growth factor alpha. *Cancer Res* **52**: 3335–3339

Bugert P and Kovacs G (1996) Molecular differential diagnosis of renal cell carcinomas by microsatellite analysis. *Am J Pathol* **149**:2081–2088

Bugert P, Gaul C, Weber K, Akhtar M, Ljungberg B and Kovacs G (1997) Specific genetic changes of diagnostic importance in chromophobe renal cell carcinoma. *Lab Invest* **76**: 203–208

Cohen T, Nahari D, Cerem LW, Neufeld G and Kevi BZ (1996) Interleukin 6 induces the expression of vascular endothelial growth factor. *J Biol Chem* **271**: 736–741

Crotty TB, Farrow GM and Lieber MM (1995) Chromophobe renal cell carcinoma: clinicopathological features of 50 cases. *J Urol* **154**: 964–967

Gogusev J, Augusti M, Chretien Y and Droz D (1993) Interleukin-6 and TNFα production in human renal cell carcinoma. *Kidney Int* **44**: 585–592

Herbers J, Schullerus D, Chudek J, Bugert P, Kanamaru H, Zeisler J, Ljungberg B, Akhtar M and Kovacs G (1998) Lack of genetic changes at specific genomic sites separates renal oncocytomas from renal cell carcinomas. *J Pathol* **184**: 58–62

Koo AS, Armstrong C, Bochner B, Shimabukuro T, Tso CL, De Kernion JB and Belldegrun A (1992) Interleukin-6 and renal cell cancer: production, regulation and growth effects. *Cancer Immunol Immunother* **35**: 97–105

Kovacs G, Akhtar M, Beckwith BJ, Bugert P, Cooper CS, Delahunt B, Eble JN, Fleming S, Ljungberg B, Medeiros LJ, Moch H, Reuter VE, Ritz E, Roos G, Schmidt D, Srigley JR, Störkel S, van den Berg E and Zbar B (1997) The Heidelberg Classification of renal cell tumours. Editorial. *J Pathol* **183**: 131–133

Lager DJ, Slagel DD and Palachek PL (1994) The expression of epidermal growth factor receptor and transforming growth factor alpha in renal cell carcinoma. *Modern Pathol* **7**: 544–548

Lahn M, Kunzmann R, Köhler G, Ikle DN, Hentrich I, Jesuiter H, Kulmburg P, Veelken H, Mackensen A, Rosenthal F and Lindemann A (1997) Comparison of cytogenetics, cytokine secretion, and oncogene expression in primary cultures of renal carcinoma cells. *Oncology* **54**: 429–437

Miki S, Iwano M, Miki Y, Yamamoto M, Tang B, Yokokawa K, Sonoda T, Hirano T and Kishimoto T (1989) Interleukin-6 (IL-6) functions as an in vitro autocrine growth factor in renal cell carcinomas. *FEBS Lett* **250**: 607–610

Palmedo G, Fischer J and Kovacs G (1997) Fluorescent microsatellite analysis reveals duplication of specific chromosomal regions in papillary renal cell tumors. *Lab Invest* **77**: 633–638

Schullerus D, Herbers J, Chudek J, Kanamaru H and Kovacs G (1997) Loss of heterozygosity at chromosomes 8p, 9p, and 14q is associated with stage and grade of non-papillary renal cell carcinomas. *J Pathol* **183**: 151–155

Stephens ND, Barton SL, Smith AY, Paul RW, Neidhart JA and Griffith JK (1996) GM-CSF secretion in primary cultures of normal and cancerous human renal cells. *Kidney Int* **50**: 1044–1050

Sunderkotter C, Steinbrink K, Goebeler M, Bhardwaj R and Sorg C (1994) Macrophages and angiogenesis. *J Leukocyte Biol* **55**: 410–422

© Cancer Research Campaign 1998